# Mitochondrial DNA keeps you young

**DOI:** 10.1038/s41419-018-1045-4

**Published:** 2018-09-24

**Authors:** Massimo Bonora, Paolo Pinton

**Affiliations:** 1 Departments of Cell Biology and Stem Cell Institute, Albert Einstein College of Medicine, Bronx, NY 10461 USA; 2 Ruth L. and David S. Gottesman Institute for Stem Cell and Regenerative Medicine Research, Albert Einstein College of Medicine, Bronx, NY 10461 USA; 3 Department of Morphology Surgery and Experimental Medicine, Section of Pathology Oncology and Experimental Biology, Laboratory for Technologies of Advanced Therapies (LTTA), University of Ferrara, Ferrara, Italy; 4 Cecilia Hospital, GVM Care and Research, 48033 Cotignola, Ravenna Italy

Many researchers have attempted to understand the mechanism of ageing and to illustrate that genetic programmes are a part of the ageing process in addition to the long-sought mechanism increasing organism entropy. A part of this programme involves the rearrangement of mitochondrial functions^1^^.^ Mitochondria are organelles that integrate cellular signaling and metabolism for carbon reduction, ATP production, and small messenger and macromolecule synthesis according to tissue demands^2^. Ageing is characterized by a decline in mitochondrial function, including a reduction in TCA cycle enzymes, a decrease in the respiratory capacity and an increase in reactive oxygen species (ROS) production, in both animal models and humans. These alterations can lead to DNA mutations, cell death, inflammation and a reduction in stem cell function, contributing to tissue degeneration. The increase in mitochondrial DNA mutations observed in aged mitochondria from both mouse models and humans is the proposed driving force^3^. Mitochondrial DNA (mtDNA) is replicated by a dedicated mitochondrial DNA polymerase (DNA pol γ), whose proofreading activity has been ablated to generate a mouse model, i.e., the so-called “mitochondrial mutator mouse”, able to introduce random mutations in mtDNA^4,5^. This model displays a strong ageing phenotype, including hair loss, graying and kyphosis, along with reduced mitochondrial respiratory complex activity and increased oxidative stress. Oxidative stress was previously considered a major determinant of ageing, and mitochondria appeared to be particularly relevant. In support of this hypothesis, antioxidant administration could partially revert the phenotype of mutator mouse-derived cells, and mice lacking the mitochondrial antioxidant enzyme SOD2 displayed an aged skin phenotype^6^. Another research group provided evidence that ROS may be dispensable for the ageing phenotype; thus, this concept remains controversial^7^.

**Fig. 1:**
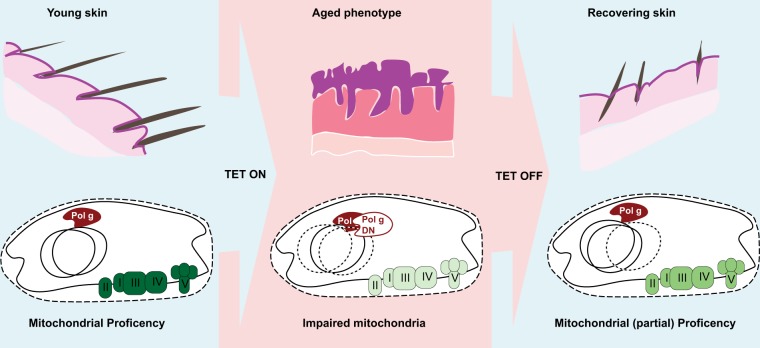
Carton is representing the mtDNA-depleter transgenic mouse model proposed by Singh and co-workers

In the last issue of Cell Death and Disease, Singh and co-workers described a novel transgenic mouse with an inducible depletion of mtDNA, i.e., the mtDNA-depleter mouse (Fig. 1)^8^. This model carries an aspartate to alanine conversion at position 1135 of POLG1 that behaves as a dominant negative for DNA pol γ, whose expression is under the control of a Tet-responsive promoter. Doxycycline administration leads to the induction of mutant DNA pol γ that blocks mtDNA replication. As mtDNA is removed by mitophagy for recycling, the activation of the transgene leads to a reduction of more than 60% in the total mtDNA content after 2 months. As mtDNA codes the core subunits of mitochondrial respiratory complexes I, III, and IV and F1/FO ATP synthase, a significant impairment was observed in their activity. Interestingly, respiratory complex II, which is only coded by nuclear genes, also displayed reduced activity. At the macroscopic level, the mtDNA-depleter mouse shows expected accelerated ageing, including weight loss and kyphosis, but ageing of the skin was particularly severe and characterized by hair loss, wrinkles and pigmentation, while at the histological level, this mouse displayed hyperplastic and hyperkeratotic epidermis, degeneration of hair follicles and extensive inflammatory infiltrates. Although the model requires extensive additional characterization, histological sections of other tested tissues (considered to have a high demand for mitochondrial activity), including the liver, brain and myocardium, do not display major alterations.

How mtDNA depletion affects ageing is a rather interesting question. The extended inflammatory infiltrates suggest that mitochondria could produce ROS as ROS can act as signaling molecules for inflammasome activation^9^; unfortunately, the author did not report measurements of oxidative stress, but cells depleted of mtDNA are usually characterized by diminished oxygen consumption and ROS production^10^, suggesting that oxidative stress should not mediate the ageing phenotype observed here. However, the following two major consequences were observed in a cell model of mtDNA depletion using the same strategy as that used in the depleter mouse: (1) a significant rearrangement of histone acetylation due to indirect alterations in the citrate levels, and (2) a reduction in cell proliferation due to a reduction in the membrane potential and destabilization of Hif1 alpha. While the type of epigenetic rearrangement that occurs during ageing is unclear, Hif1a depletion has been shown to lead to an accelerated aged skin phenotype in mice^11^. Additionally, skin mitochondria with depleted DNA display a loss of mitochondrial cristae presumably due to the loss of F1/FO ATP synthase dimers. Indeed, the rearrangement of ATP synthase dimers could predispose cells to death by opening the permeability transition pore channel (PTPC)^12^. The authors did not report a measure of cell death (that was observed in the mutator mouse); nonetheless, since multiple mitochondrial molecules can act as damage-associated molecular patterns (DAMPs), an increase in PTPC-mediated cell death could cause the extensive release of mito-DAMP and, ultimately, the observed increase in inflammation.

Another extremely interesting point in this study is the recovery of the phenotype. Halting doxycycline exposure led to a surprising and almost complete recovery of the mtDNA content and skin phenotype after one month. The recovery of the mtDNA content is expected since the original mtDNA was not completely exhausted. The recovery of the skin phenotype is more intriguing. The mutator mouse model provided important insight into how mitochondria can induce an ageing phenotype by affecting haematopoietic and neural stem cell self-renewal capacities^13,14^. We speculate that mtDNA depletion affects epidermal stem cell function, leading to skin ageing. Although it has long been thought that stem cells do not rely on mitochondrial function (at least for ATP production), additional observations in adult stem cells from other tissues suggest that mitochondria can be fundamental for stem cell self-renewal^15^. However, progenitor cells, which have an established dependency on mitochondrial respiration in many models, could be more sensitive to mtDNA depletion and therefore responsible for the rapid recovery.

Frequently, a new observation raises multiple questions. Even if further investigation is required, the mouse model developed by Singh and co-worker is an extremely interesting tool for investigating ageing and the role of mitochondria in tissue homeostasis.
